# Standardized Preparation of Single-Cell Suspensions from Mouse Lung Tissue using the gentleMACS Dissociator

**DOI:** 10.3791/1266

**Published:** 2009-07-02

**Authors:** Melanie Jungblut, Karen Oeltze, Irene Zehnter, Doris Hasselmann, Andreas Bosio

**Affiliations:** Miltenyi Biotec,GmbH

## Abstract

The preparation of single-cell suspensions from tissues is an important prerequisite for many experiments in cellular research. The process of dissociating whole organs requires specific parameters in order to obtain a high number of viable cells in a reproducible manner. The gentleMACS Dissociator optimizes this task with a simple, practical protocol. The instrument contains pre-programmed settings that are optimized for the efficient but gentle dissociation of a variety of tissue types, including mouse lungs. In this publication the use of the gentleMACS Dissociator on lung tissue derived from mice is demonstrated.

**Figure:**
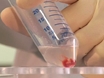


## Protocol

To work under sterile conditions, it is recommended to perform all steps in a laminar flow hood.

### 1. Materials

HEPES buffer: 10 mM HEPES-NaOH pH 7.4, 150 mM NaCl, 5 mM KCl, 1 mM MgCl_2_, 1.8 mM CaCl_2_)
Collagenase D solution: Collagenase D: 100 mg/mL (Collagenase D > 0.15 U/mg), in HEPES buffer
DNase I solution: 20,000 U/mL DNase I
PBS buffer at pH 7.2
PEB buffer: 1 part BSA Stock Solution in 20 parts autoMACS Rinsing Solution
Tissue: lung derived from 6/7 week old adult female BALB/C mouse, free of adjoining organs.
Optional: CD11c MicroBeads, MACS MS Columns, MACS Separator for the isolation of dendritic cells from mouse lung single-cell suspension

### 2. Dissociating the lung tissue

Rinse tissue in a Petri dish containing PBS to remove erythrocytes.
Transfer a maximum of 450 mg lung tissue to a gentleMACS C Tube containing 4.9 mL HEPES buffer.
Add 100 μL Collagenase D solution to a final concentration of 2 mg/ml Collagenase D.
Add 10 μL DNase I solution for up to 150 mg tissue (final concentration of 40 U/ml DNase I) or 20 μL DNase I for 150-450 mg lung tissue (final concentration of 80 U/mL DNase I).
Tightly close the C Tube and attach it onto the gentleMACS Dissociator. Then run the program "m_lung_01".
Incubate for 30 min at 37°C with automated rotation or manually turning every 5 min.
Return sample to the gentleMACS Dissociator and run the program "m_lung_02".

### 3. Filtration

Prepare a 50 mL tube for collecting filtered cells by replacing the cap with a 70 μm mesh cell strainer.
Once the second gentleMACS Program ends, depending on the distribution of sample material in the tube, you may centrifuge the tube briefly to collect the sample material at the bottom of the tube.
Remove the cells through the septum sealed cap of the C Tube using a suitable 1000 μl pipette tip and apply them to the cell strainer.
Wash the cell strainer with 5 ml HEPES buffer at RT.
Centrifuge the cells to a pellet in a 50 mL tube at 300xg for 10 min.
Aspirate the supernatant and resuspend the cell pellet in your desired volume of PEB buffer.

### Part 4: Representative Results:


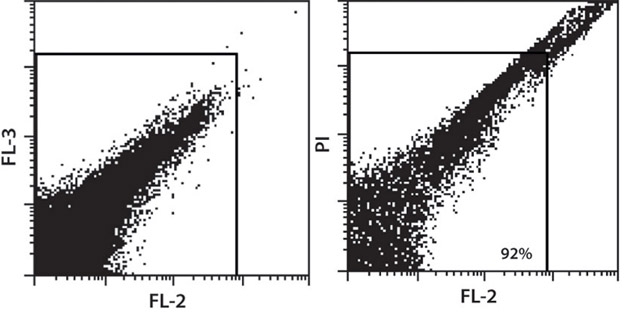
 **Figure 1.** Lung dissociation with the gentleMACS™ Dissociator resulted in 92% viable cells. Dead cells were fluorescently stained with propidium iodide (PI) ( right dot plot; left dot plot: no PI staining).


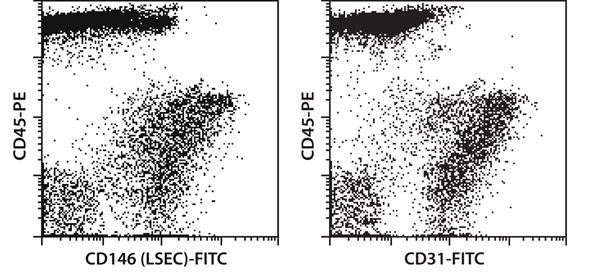
 **Figure 2.** Dissociation of mouse lung tissue using the gentleMACS Dissociator results in a high percentage of viable leukocytes and endothelial cells. The derived single-cell suspensions were stained with CD45-PE and CD146-FITC or CD31-FITC and analyzed by flow cytometry.


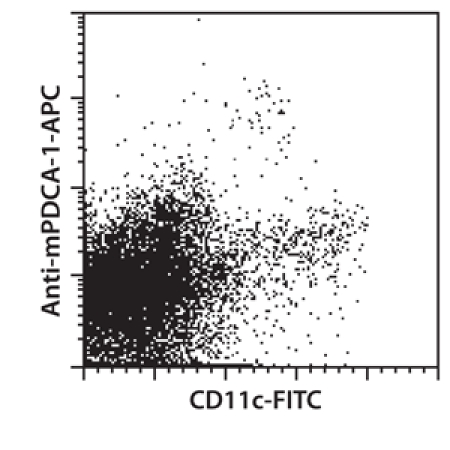
 **Figure 3. **Single-cell suspension derived from mouse lung tissue was stained with CD11c-FITC and Anti-mPDCA-1-APC to detect mouse CD11c^low^ m-PDCA-1+ plasmacytoid DCs as well as CD11c^high^ cells.


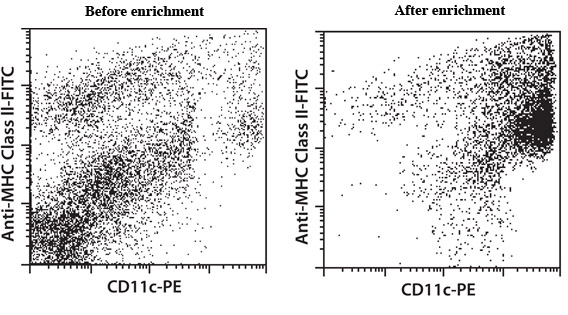
 **Figure 4. ** Enrichment of dendritic cells using CD11c MicroBeads, a MiniMACS Separator and two MS Columns.

## Discussion

In this video, we introduce a new method for the dissociation of mouse lung tissue. We show that a combination of mechanical and enzymatic treatment of lung tissue yielded a high percentage of viable leukocytes and endothelial cells. Specifically, the mechanical disintegration of the tissue was achieved by the gentleMACS Dissociator. The gentleMACS C Tubes include a rotor - stator system, which dissociates tissue in a gentle way. The procedure is controlled by the program settings of the instrument. The settings were optimized to achieve high yield and viability of cells. The derived single-cell suspensions were stained with CD45-PE and CD146-FITC or CD31-FITC and analyzed by flow cytometry to detect leukocytes and endothelial cells. Dendritic cells in the single-cell suspension were stained with CD11c-FITC and Anti-mPDCA-1-APC. Furthermore, dendritic cells from a mouse lung single-cell suspension were enriched using MACS Technology: dendritic cells were magnetically labeled using CD11c MicroBeads and separated using a MiniMACS Separator and MS Columns. ^1,2^ In general, the combination of our new dissociation protocol with magnetic cell sorting represents a standardized method to obtain specific cell types from lung tissue.
